# Nano-Crystallization of Ln-Fluoride Crystals in Glass-Ceramics via Inducing of Yb^3+^ for Efficient Near-Infrared Upconversion Luminescence of Tm^3+^

**DOI:** 10.3390/nano11041033

**Published:** 2021-04-18

**Authors:** Jianfeng Li, Yi Long, Qichao Zhao, Shupei Zheng, Zaijin Fang, Bai-Ou Guan

**Affiliations:** Guangdong Provincial Key Laboratory of Optical Fiber Sensing and Communications, Institute of Photonics Technology, Jinan University, Guangzhou 511443, China; ljf151@stu2019.jnu.edu.cn (J.L.); longyi9604@stu2019.jnu.edu.cn (Y.L.); a1290816237@stu2020.jnu.edu.cn (Q.Z.); tguanbo@jnu.edu.cn (B.-O.G.)

**Keywords:** nano-crystallized glass ceramic, nano-crystallization, luminescence, Tm^3+^ doped, upconversion

## Abstract

Transparent glass-ceramic composites embedded with Ln-fluoride nanocrystals are prepared in this work to enhance the upconversion luminescence of Tm^3+^. The crystalline phases, microstructures, and photoluminescence properties of samples are carefully investigated. KYb_3_F_10_ nanocrystals are proved to controllably precipitate in the glass-ceramics via the inducing of Yb^3+^ when the doping concentration varies from 0.5 to 1.5 mol%. Pure near-infrared upconversion emissions are observed and the emission intensities are enhanced in the glass-ceramics as compared to in the precursor glass due to the incorporation of Tm^3+^ into the KYb_3_F_10_ crystal structures via substitutions for Yb^3+^. Furthermore, KYb_2_F_7_ crystals are also nano-crystallized in the glass-ceramics when the Yb^3+^ concentration exceeds 2.0 mol%. The upconversion emission intensity of Tm^3+^ is further enhanced by seven times as Tm^3+^ enters the lattice sites of pure KYb_2_F_7_ nanocrystals. The designed glass ceramics provide efficient gain materials for optical applications in the biological transmission window. Moreover, the controllable nano-crystallization strategy induced by Yb^3+^ opens a new way for engineering a wide range of functional nanomaterials with effective incorporation of Ln^3+^ ions into fluoride crystal structures.

## 1. Introduction

Trivalent lanthanide (Ln^3+^) ions doped upconversion (UC) luminescent materials have been extensively investigated for applications in lighting, solar cells, solid-state laser, biological imaging, and temperature sensing due to the multiple luminescence wavelength from ultraviolet to near-infrared (NIR) regions [1,2,3,4,5,6,7,8,9]. Among these, Tm^3+^ doped materials possess UC luminescence in the NIR region at around 800 nm (^3^H_4_→^3^H_6_), which is located in the biological transmission window, and the light in that region can easily penetrate biological tissues [10,11,12,13]. Moreover, the UC emissions of Tm^3+^ are dramatically enhanced via the energy transfer (ET) from Yb^3+^, which possesses a large absorption section for the commercial 980 nm lasers. Thus, Yb^3+^-Tm^3+^ co-doped materials were more efficient candidates for NIR-to-NIR UC luminescence and were significant light sources for biologically non-destructive detection. In the past decades, a large number of investigations about Yb^3+^-Tm^3+^ co-doped UC materials have been widely reported for achieving high-efficiency NIR UC luminescence [14,15,16,17,18,19,20].

Glass exhibits high transmittance, easy fabrication, and excellent processability due to its amorphous and softening characteristics. These properties make glass an appealing matrix for the design and fabrication of special optics devices, particularly of optical fiber lasers [21,22], which can hardly be achieved by using crystals. Furthermore, nano-crystallized glass ceramic (GC) composites can be obtained via the precipitation of functional nanocrystals in a glass matrix via heat treatments. The nano-crystallized GCs still possess high optical transmittance and the UC luminescence efficiency in GCs can be greatly enhanced, as compared to glass, when Ln^3+^ ions are successfully incorporated into the crystal structures featuring lower probability of non-radiative transition ascribed to the lower phonon energy. So far, Yb^3+^-Tm^3+^ co-doped GCs have been considered as significantly optical gain materials for UC NIR fiber lasers, noncontact optical thermometers, and bio-imaging [23,24,25,26]. Generally, Ln^3+^ ions were expected to incorporate into the crystal structures via the ionic substitution for Y^3+^, La^3+^, Lu^3+^, Sc^3+^, and Sr^2+^ in the GCs embedded with Na(Y/La/Lu)F_4_, LaF_3_, YF_3_, KSc_2_F_7_, and SrF_2_ crystals, etc [27,28,29,30,31]. However, the ionic substitution process was uncontrollable and the amount of Ln^3+^ incorporated into the crystal structures was usually small due to the large mismatch between Ln^3+^ and the replaceable sites. The enhancements of luminescence in GCs were limited. Moreover, a lot of remaining crystals not occupied by Ln^3+^ ions made no contribution to the enhancement of luminescence but will trigger severe issues of optical scattering and low optical transmittance in glass. These made the traditional GCs difficult to be used in the practical applications. Accordingly, it is highly desirable for design and fabrication of novel GCs for controllably incorporating a large number of Ln^3+^ into crystal structures to achieve high-efficiency NIR UC luminescence of Tm^3+^.

Ln-fluoride crystal, intrinsically containing Ln^3+^ (for example, Yb^3+^ or Er^3+^) in the crystal lattices, is a significant host for the incorporation of other active Ln^3+^ ions because the mismatches in ionic radius between different Ln^3+^ ions are smaller and active Ln^3+^ ions enter Ln-fluoride crystals more easily [32]. In this work, GCs containing Ln-fluoride nanocrystals were designed to greatly enhance the UC luminescence of Tm^3+^ and achieve pure, high-efficiency NIR-to-NIR emissions. Two kinds of Ln-fluoride nanocrystals (KYb_3_F_10_ and KYb_2_F_7_) were controllably precipitated from a glass matrix via the heat treatments dependent on the doping of Yb^3+^. Tm^3+^ ions entered the fluoride crystal lattices easily by replacing Yb^3+^ sites. As a result, a large number of Ln^3+^ were incorporated into the fluoride crystals featuring extremely low phonon energy and the UC emission of Tm^3+^ in the GCs were dramatically enhanced. Moreover, the pure NIR UC emissions were also obtained by adjusting the doping concentration of Tm^3+^. Thus, the designed GCs provide an efficient material for pure NIR UC luminescence and offer highly promising developments for UC fiber lasers, non-contact optical thermometers, and bio-imaging.

## 2. Materials and Methods

### 2.1. Materials Preparation

Samples with a molar composition of 70SiO_2_-15KF-15ZnF_2_-*x*YbF_3_-*y*Tm_2_O_3_ (*x* = 0~3.5, *y* = 0~0.3) were prepared using a melt quenching method. First, 30 g reagent grade stoichiometric mixtures were mixed thoroughly in an agate mortar and melted in covered quartz crucibles in an electric furnace at 1550 °C for 30 min; the melts were poured onto a brass plate and then pressed using another brass plate to obtain precursor glasses (PGs). PG samples were heated at 540 °C for 5 and 10 h to obtain GC samples according to the differential scanning calorimetry (DSC) results in ref. [33]. PG and GC samples were cut and polished to 2 mm thick for measurements.

### 2.2. Characterizations

To identify the crystalline phase in GCs, X-ray diffraction (XRD) patterns were performed on a X-ray diffractometer (Bruker, Fällanden, Switzerland) with Cu/Ka (λ = 0.1541 nm) radiation. The morphology and size distribution of the nanocrystals in GCs were measured via high-resolution transmission electron microscopy (HRTEM) (FEI, Hillsboro, OR, USA). UC emission spectra of samples were recorded using an Edinburgh FLS980 fluorescence spectrometer (Edinburgh Instruments, Edinburgh, UK). A 980 nm laser diode (LD) was used as the exciting source for the measurement of UC emission spectra. The emission decay curves were measured using the same spectrometer with a microsecond lamp as the excitation source. All measurements were performed at room temperature.

## 3. Results and Discussion

**Figure 1:**
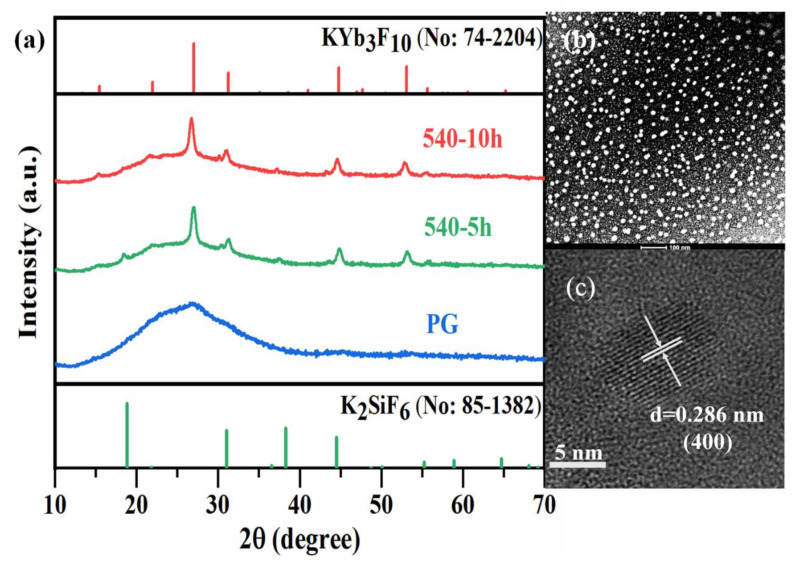
(**a**) XRD patterns of 1.0Yb^3+^-0.005Tm^3+^ co-doped PG and GCs. (**b**) TEM and (**c**) HRTEM images of 1.5Yb^3+^-0.1Tm^3+^ co-doped GC heat treated at 540 °C for 10 h.

Figure 1a shows the XRD patterns of Yb^3+^-Tm^3+^ co-doped PG and GCs. A broad band is observed in the XRD pattern of PG due to the amorphous characteristic of glass. Sharp peaks are observed in the XRD patterns of the two GCs. The main peaks at 26.99°, 31.27°, 44.81°, and 53.1° are ascribed to the (222), (400), (440), and (622) crystal facets of KYb_3_F_10_ (No: 74-2204), respectively, indicating that KYb_3_F_10_ crystals have been precipitated in the GCs. Moreover, weak peaks at 18.86°, 31.04°, and 38.28° attributed to the diffraction peaks of K_2_SiF_6_ (No: 85-1382) crystals are also observed in the XRD patterns of GC heated at 540 °C for 5 h. It is also found that the intensities of diffraction peaks for KYb_3_F_10_ crystal are all increased when the heating time increases from 5 to 10 h. However, the diffraction peaks of K_2_SiF_6_ crystal all weaken due to the further heat treatment to 10 h. These results indicate that KYb_3_F_10_ crystals prefer to precipitate in the 1.0Yb^3+^-0.005Tm^3+^ co-doped GCs via the further heat treatment. Additionally, the average size of crystals can be calculated by the Scherrer’s equation. The diffraction peak, around 2θ = 27.01° and 18.42°, was selected for the calculation, and the average size of the KYb_3_F_10_ and K_2_SiF_6_ nanocrystals in the GC heated at 540 °C for 5 h was calculated to be approximately 13.35 and 31.34 nm, respectively.

The HAADF-TEM image shown in Figure 1b reveals that the nanocrystals are in-situ, precipitated among the glass matrix. The measured size of these nanoparticles is from 10 to 30 nm. The HR-TEM image is shown in Figure 1c. The crystal lattice fringes are obvious, which is different from that of the amorphous glass matrix. The interval of the crystal lattice fringes d can be measured directly, and its value is about 0.286 nm, which corresponds to the (400) crystal facet of cubic KYb_3_F_10_. These also prove that KYb_3_F_10_ nanocrystals are precipitated in the GCs.

**Figure 2:**
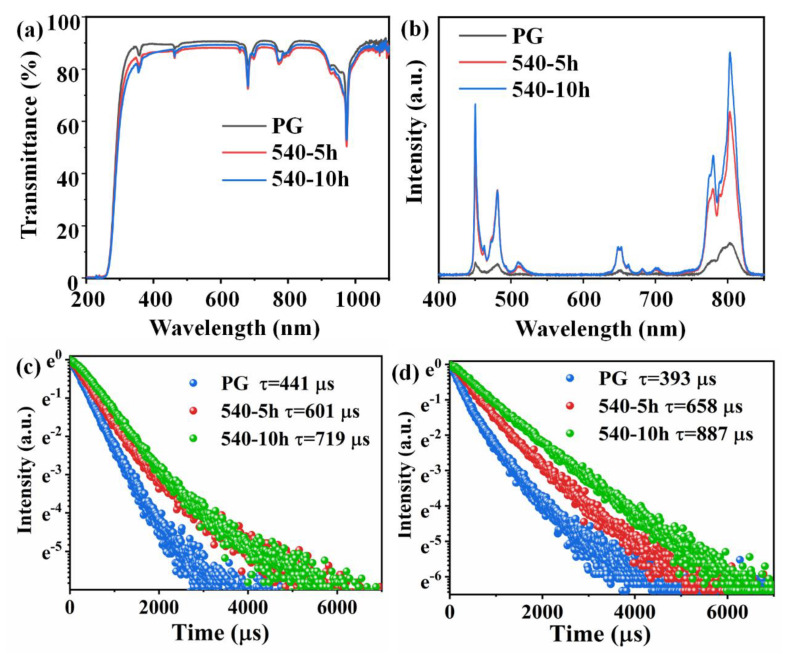
(**a**) Transmission spectra of 1.0Yb^3+^-0.1Tm co-doped PG and GC. (**b**) UC emission spectra 1.0Yb^3+^-0.005Tm^3+^co-doped PG and GCs. (**c**) Emission decay curves of 1.0Yb^3+^-0.005Tm^3+^ co-doped PG and GCs monitored at 450 nm emission. (**d**) Emission decay curves of 1.0Yb^3+^-0.005Tm^3+^ co-doped PG and GCs monitored at 802 nm emission.

The optical transmission spectra of the 1.0Yb^3+^-0.1Tm^3+^ co-doped samples are shown in Figure 2a. The PG sample possesses high transmittance (~90%) from 300 to 800 nm with a thickness of 2 mm. Though nanocrystals are precipitated in the GCs, the optical transmittances of GCs are still as high as 85%. Interestingly, the transmittance of the GC heated for 10 h is higher than that heated for 5 h. As calculated from the XRD patterns, the average size of KYb_3_F_10_ crystals is smaller than that of K_2_SiF_6_ crystals. When the heat treatment time increases from 5 to 10 h, more KYb_3_F_10_ crystals and less K_2_SiF_6_ crystals are precipitated in the GC, resulting in the increase of transmittance.

Figure 2b shows the emission spectra of 1.0Yb^3+^-0.005Tm^3+^ co-doped PG and GCs recorded at room temperature upon the excitation of a 980 nm laser diode (LD). The spectra all include a NIR emission peak at 802 nm attributed to the ^3^H_4_→^3^H_6_ transition of Tm^3+^. Meanwhile, the blue (450 and 480 nm), red (650 nm), and deep red emission peaks (680 and 700 nm) attributed to ^1^D_2_→^3^F_4_, ^1^G_4_→^3^H_6_, ^1^G_4_→^3^F_4_, ^3^F_2_→^3^H_4_, and ^3^F_3_→^3^H_4_ transition of Tm^3+^ [34,35], respectively, are all observed in the emission spectra of PG and GC samples. Since Tm^3+^ exhibits no absorption for 980 nm light, these emissions are all obtained via the ET processes from Yb^3+^ to Tm^3+^. In addition, the emission peaks of GCs are narrower than that of PG and the splitting of spectrum by crystal fields are observed in the spectra of GCs, indicating the incorporation of Tm^3+^ into crystal coordinated sites. It is also found from the spectra that the emission intensity at 450 and 802 nm increases 15 and 6 times after the heat treatment for 10 h, respectively. Figure 2c,d show the emission decay curves monitored at 450 and 802 nm, respectively. The emission lifetime at 450 nm increases from 441 to 601 and 719 μs, and that at 802 nm increases from 393 to 658 and 887 μs when the sample is heated at 540 °C for 5 and 10 h, respectively. These results are attributed to the fact that the low phonon energy environment reduces the possibility of non-radiative transition and enhances the emission intensity and lifetime in the sample, which in turn prove the incorporation of Tm^3+^ into the fluoride nanocrystals in GCs. Actually, it is difficult for Tm^3+^ to enter K_2_SiF_6_ crystal structures due to the lack of appropriate sites for the substitution of Tm^3+^. However, Tm^3+^ can easily incorporate into KYb_3_F_10_ crystal structures by occupying the sites of Yb^3+^ because the ionic radiuses of Tm^3+^ (R = 0.086 nm) and Yb^3+^ (R = 0.085 nm) are very similar. Accordingly, the incorporation of Tm^3+^ into KYb_3_F_10_ nanocrystals is responsible for the enhancements of emission intensities and lifetimes in GCs.

**Figure 3:**
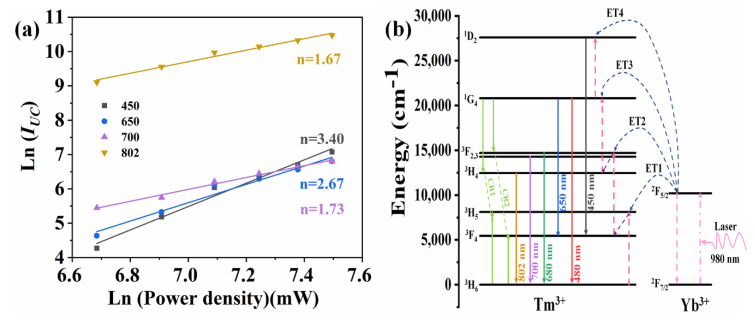
(**a**) Ln-Ln plots of UC emission intensity versus excited power density of 980 nm laser. (**b**) Schematic diagrams of energy levels and relative transitions in KYb_3_F_10_:Tm^3+^ GCs.

The double-logarithmic plots of the excitation power dependency on the emission intensities are presented in Figure 3a. The fitted slope (n) of the plot of Ln (I_UC_) versus Ln (Power) is used to determine the absorbed photon numbers per UC emitted [36,37]. The obtained value of n was 1.67, 1.73, 2.67, and 3.40 corresponding to the 802,700, 650, and 450 nm emission of 1.0Yb^3+^-0.005Tm^3+^ co-doped GC, respectively. These prove that the emissions are all attributed to UC emission of Tm^3+^ and obtained via the ET from Yb^3+^. Two, two, three, and four pump photons are needed to pump the electrons to ^3^H_4_, ^3^F_2,3_, ^1^G_4_, and ^1^D_2_ energy levels of Tm^3+^ to achieve the corresponding UC emissions, respectively.

Based on these, the energy levels of Tm^3+^ and Yb^3+^ and the relative transitions corresponding to the above UC emissions are illuminated in Figure 3b [38,39,40]. Under 980 nm LD excitation, the electrons of Yb^3+^ are excited from the ground state ^2^F_7/2_ to the ^2^F_5/2_ excited state, then the energy is transferred to Tm^3+^ through the ET1 process: [^2^F_5/2_ (Yb^3+^) + ^3^H_6_ (Tm^3+^)→^2^F_7/2_ (Yb^3+^) + ^3^H_5_ (Tm^3+^) + phonons]. The electrons of Tm^3+^ are excited from the ground state to the ^3^H_5_ excited state and then undergo a non-radiative relaxation transition to the ^3^F_4_ energy level. Via the ET2 process, [^2^F_5/2_ (Yb^3+^) + ^3^F_4_ (Tm^3+^)→^2^F_7/2_ (Yb^3+^) + ^3^F_2,3_ (Tm^3+^) + phonons], the electrons are populated to the ^3^F_2, 3_ energy level. A part of the electrons undergo a non-radiative relaxation transition to ^3^H_4_ energy level. In this process, ^3^H_4_→^3^H_6_ (802 nm) and ^3^F_2,3_→^3^H_6_ (700 and 680 nm) transitions are obtained. Next, part of the electrons at ^3^H_4_ energy levels are excited to ^1^G_4_ energy through the process of ET3: [^2^F_5/2_ (Yb^3+^) + ^3^H_4_ (Tm^3+^)→^2^F_7/2_ (Yb^3+^) + ^1^G_4_ (Tm^3+^) + phonons]. This produces ^1^G_4_→^3^H_6_ (480 nm) and ^1^G_4_→^3^F_4_ (650 nm) transitions. Finally, the electrons of the ^1^G_4_ energy level are populated to the ^1^D_2_ energy level through the process of ET4, [^2^F_5/2_ (Yb^3+^) + ^1^G_4_ (Tm^3+^)→^2^F_7/2_ (Yb^3+^) + ^1^D_2_ (Tm^3+^) + phonons], producing a ^1^D_2_→^3^F_4_ (450 nm) transition.

**Figure 4:**
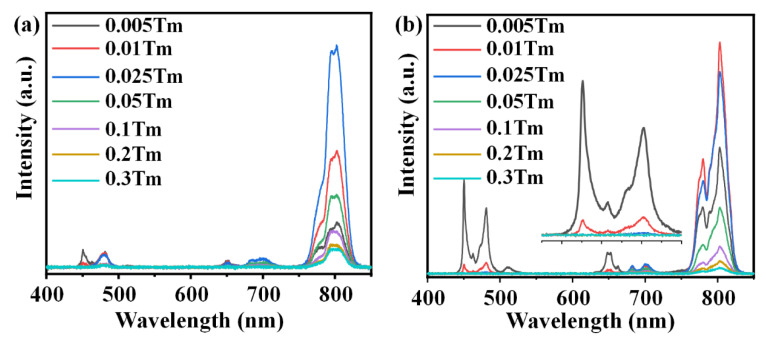
UC emission spectra of 1.0Yb^3+^-*y*Tm^3+^ co-doped (**a**) PG and (**b**) GC excited by a 980 nm LD. (*y* = 0.005~0.3).

Furthermore, the transitions of Tm^3+^ are also modulated via the interaction between neighboring Tm^3+^ ions, which is directly determined by the doping concentration of Tm^3+^ ions [41,42]. The dependence of UC emission spectrum on the doping concentration of Tm^3+^ in the co-doped PG and GC is presented in Figure 4a,b. It can be observed from the spectra that the blue emission intensities at 450 nm and 480 nm both decrease monotonically with the increase of Tm^3+^ concentration, while the NIR emission intensity at 802 nm experiences first an enhancement and then a decrease due to the further increase of Tm^3+^ concentration. Actually, as a result of the increase of Tm^3+^ content, the distance between Tm^3+^ becomes shorter. The interactions between neighboring Tm^3+^ ions get larger, which benefits the cross-relaxation (CR) process, ^1^G_4_ + ^3^H_6_→^3^F_2,3_ + ^3^F_4_, as shown in Figure 3b. The CR process depopulates electrons in the ^1^G_4_ level, while the electrons in ^3^F_2,3_ and ^3^F_4_ levels are populated via the CR process. Thus, blue emission intensities decrease quickly with the increase of Tm^3+^ even though the doping concentration is low. The NIR emission increases firstly and then decreases when the doping concentration is further increased due to the concentration quenching effect. Compared to a glass matrix, crystal exhibits a more compact structure. The distance between Tm^3+^ in GC is shorter than that in PG at the same doping concentration, resulting in the lower luminescence-quenching concentration in GC as compared to PG. The emission intensity at 802 nm reaches a maximum with the doping of 0.025 and 0.01Tm^3+^ in PG and GC, respectively. As shown in Figure 4b and the inset, the blue emissions around 450 and 480 nm in PG and GC both disappear and the UC spectra almost exhibit pure NIR emissions when the contents of Tm^3+^ exceed 0.01 mol%. A blue UC emission only being obtained in GC at a low doping concentration indicates most Tm^3+^ ions are incorporated into the KYb_3_F_10_ crystal structure with short interionic distance. The pure NIR emissions that are obtained at a very low doping concentration of Tm^3+^ indicates that the GC is an excellent candidate for pure NIR UC emission and is a significant UC luminescent material for promising applications in NIR fiber lasers and high-resolution biological imaging.

**Figure 5:**
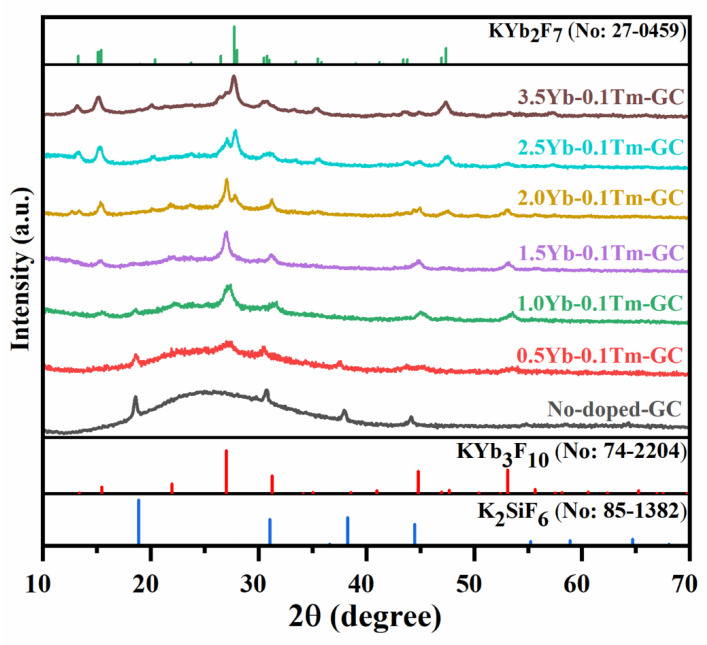
XRD patterns of *x*Yb^3+^-0.1Tm^3+^co-doped and no-doped GCs heat treated at 540 °C for 10 h. (*x* = 0~3.5%).

As mentioned above, Yb^3+^ works as sensitizer ion for the UC emissions of Tm^3+^. More importantly, Yb^3+^ also participates in the construction of fluoride nanocrystals and provides appropriate crystal sites for the incorporation of Tm^3+^. The doping of Yb^3+^ plays a vital role in the precipitation of nanocrystals and enhancement of the UC emissions. The Yb^3+^-concentration-dependent XRD patterns of the Yb^3+^-Tm^3+^ co-doped and no-doped GCs are presented in Figure 5. It is found that only K_2_SiF_6_ crystals are precipitated in the no-doped GC and these make no contribution to the enhancement of UC emission. Via the doping of Yb^3+^, KYb_3_F_10_ are precipitated in the GCs apart from K_2_SiF_6_ crystals as the Yb^3+^ content increases from 0.5 to 1.0 mol%. When Yb^3+^ concentration is set to 1.5 mol%, only the diffraction peaks of KYb_3_F_10_ crystals are observed in the XRD pattern of GC. These results indicate that the precipitation of KYb_3_F_10_ nanocrystals in the GCs is governed by the doping concentration of Yb^3+^ induced by Yb^3+^. More interestingly, KYb_2_F_7_ crystals are also precipitated in the GCs when the Yb^3+^ concentration is increased from 2.0 to 3.5 mol%. In the XRD pattern of the 3.5Yb^3+^-0.1Tm^3+^ co-doped sample, only the diffraction peaks of KYb_2_F_7_ crystals are observed. Therefore, the nano-crystallization in GC is induced by the doped Yb^3+^ and the crystalline phase in the GC can be controllably regulated from K_2_SiF_6_ to KYb_3_F_10_ and further changes to KYb_2_F_7_ crystals by adjusting the concentration of Yb^3+^ from 0 to 3.5 mol%.

**Figure 6:**
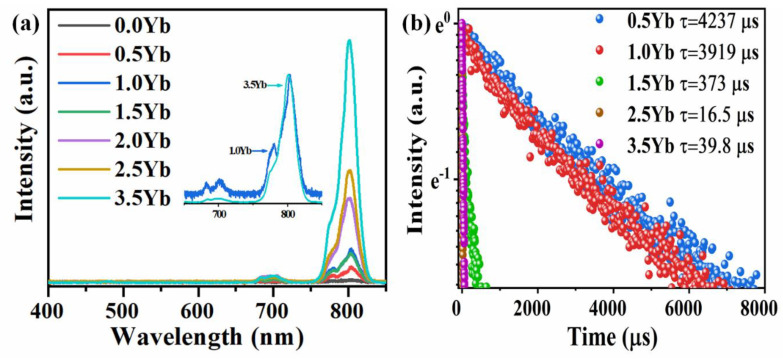
(**a**) UC emission spectra *x*Yb^3+^-0.1Tm^3+^co-doped GCs heat treated at 540 °C for 5 h. (*x* = 0.0~3.5). (**b**) Emission decay curves of *x*Yb^3+^-0.1Tm^3+^ co-doped PG and GCs heat treated at 540 °C for 5 h monitored at 802 nm emission. (*x* = 0.5~3.5).

Via the nano-crystallizations of KYb_3_F_10_ and KYb_2_F_7_, Yb^3+^ ions are spontaneously confined within fluoride crystal structures. More importantly, Tm^3+^ can easily incorporate these two crystal structures by the substitution of Yb^3+^. Owing to the extremely low phonon energies in the fluoride crystals, the probabilities of non-radiative transitions are low, which is beneficial for the efficient ET from Yb^3+^ to Tm^3+^ and enhanced UC emission of Tm^3+^. The UC emission spectra of Yb^3+^-Tm^3+^ co-doped GC with various concentrations of Yb^3+^ are shown in Figure 6a. Excited by a 980 nm LD, pure NIR UC emissions around 802 nm of Tm^3+^ are observed in the spectra of GCs. As the Yb^3+^ concentration increases from 0.5 to 1.5 mol%, the quantity of KYb_3_F_10_ nanocrystals precipitated in the GCs is increased, and the amount of Tm^3+^ and Yb^3+^ ions located in the fluoride crystal structures are increased. The emission intensity enhances firstly, reaching a maximum at 1.0 mol% Yb^3+^, and then decreases when the Yb^3+^ concentration is increased to 1.5 mol% due to the concentration quenching effect caused by the severe interactions between Ln^3+^ ions. When the Yb^3+^ concentration is further increased to 2.0 mol%, KYb_2_F_7_ crystals start to precipitate in the GCs. A part of the Tm^3+^ enter KYb_2_F_7_ nanocrystals in the 2.0Yb^3+^-0.1Tm^3+^ co-doped GC, which disperses the distribution of Tm^3+^ and increases the NIR UC emission intensity again. When the Yb^3+^ concentration increases from 2.0 to 3.5 mol%, more KYb_2_F_7_ crystals are precipitated in the GCs, more Tm^3+^ ions are incorporated into KYb_2_F_7_ crystals, and the UC emission intensity at 800 is increased monotonically. It is also found that the profile of the emission spectrum for the 3.5 Yb^3+^-0.1Tm^3+^ co-doped GCs is different from that of the 1.0Yb^3+^0.1Tm^3+^ co-doped samples (inset of Figure 6a), which in turn proves that Tm^3+^ are incorporated in two different crystal environments in the two GCs containing different nanocrystals. Compared to KYb_2_F_7_ crystal, more Yb^3+^ ions are distributed in the crystal structure in KYb_3_F_10_. The luminescent-quenching concentration of the UC emission in KYb_2_F_7_ crystal is higher than that in KYb_3_F_10_. The emission intensity in 3.5Yb^3+^-0.1Tm^3+^ co-doped GC is 7 times higher than that in the 1.0Yb^3+^-0.1Tm^3+^ sample, which indicates that the nano-crystallization of KYb_2_F_7_ provides more excellent crystal environments for achieving more efficient NIR UC emission of Tm^3+^.

The UC emission decay curves of the Yb^3+^-Tm^3+^ co-doped GCs monitored at 802 nm are shown in Figure 6b. The lifetime of the NIR emission decreases as Yb^3+^ concentration increases from 0.5 to 1.5 mol%. Especially, the lifetime of the 1.5Yb^3+^-0.1Tm^3+^ co-doped GC decreases dramatically to 373 μs due to the severe interaction between the neighboring Tm^3+^ in KYb_3_F_10_ crystals. However, the lifetime increases from 16.5 to 39.8 μs when the Yb^3+^ concentration is increased from 2.5 to 3.5 mol%. These results also prove that the coordinated environments of Tm^3+^ in high-doping GCs (2.5 and 3.5 mol% Yb^3+^-0.1Tm^3+^) is distinct to that in low-doping GCs (0.5, 1.0, and 1.5 mol% Yb^3+^-0.1Tm^3+^). Therefore, the precipitation of KYb_2_F_7_ nanocrystals in GCs further enhances the NIR UC emission of Tm^3+^ and provides a significant matrix for applications in photonic devices, in particular of high-efficiency UC fiber lasers.

## 4. Conclusions

In summary, transparent Yb^3+^-Tm^3+^ co-doped GCs were prepared in this work for greatly enhancing the UC luminescence of Tm^3+^. The GCs possessed high transmittance (>85.0%) though fluoride nanocrystals were precipitated among the glass matrices. K_2_SiF_6_ crystals are precipitated in the no-doped GC. However, KYb_3_F_10_ and KYb_2_F_7_ nanocrystals were controllably and successively precipitated in the GCs via the inducing of Yb^3+^ when the doping concentration of Yb^3+^ changed from 0.5 to 3.5 mol%. UC emissions of Tm^3+^ were dramatically enhanced when Ln^3+^ ions were incorporated into KYb_3_F_10_ crystal structures as the Yb^3+^ concentration changed from 0.5 to 1.5 mol%. Pure NIR UC emissions were obtained by adjusting the concentration of Tm^3+^. KYb_2_F_7_ nanocrystals were precipitated in GCs when the Yb^3+^ concentration exceeded 2.0 mol%. More efficient UC emissions were achieved via the precipitation of KYb_2_F_7_ than that of KYb_3_F_10_ in GCs. The designed GCs offer potential optical gain materials for highly-efficient NIR UC photoluminescence. More importantly, the Yb^3+^-induced nano-crystallization strategy paves a new way for the design and fabrication of emerging GCs to provide excellent crystal environments for Ln^3+^.
